# The challenges of pig farming in Hong Kong: a study of farmers’ perceptions and attitudes towards a pig health and production management service

**DOI:** 10.1186/s12917-023-03591-7

**Published:** 2023-02-01

**Authors:** Sarah M. Rosanowski, Ioannis Magouras, Wing-Chung Ho, Wing Chi Jacqueline Yiu, Dirk U. Pfeiffer, Friederike Zeeh

**Affiliations:** 1 Centre for Applied One Health Research and Policy Advice, Jockey Club College of Veterinary Medicine and Life Sciences, City University of Hong Kong, Kowloon, Hong Kong SAR, China; 2 Digital Agriculture, Grasslands Research Centre, AgResearch Limited, Palmerston North, New Zealand; 3 Department of Infectious Diseases and Public Health, Jockey Club College of Veterinary Medicine and Life Sciences, City University of Hong Kong, Kowloon, Hong Kong SAR, China; 4 Department of Social and Behavioural Sciences, College of Liberal Arts and Social Sciences, City University of Hong Kong, Kowloon, Hong Kong SAR, China

**Keywords:** Attitudes, Barriers, Veterinary service, Qualitative approach, Mixed methods, Pig farming

## Abstract

**Background:**

Pig farming in Hong Kong differs markedly from other places in the world, with a highly urbanised population, the majority of pigs being imported for slaughter, and limited on-farm veterinary support. Little is known about the barriers and attitudes of pig farmers in Hong Kong and their expectations of a new pig health and production management service provided by veterinarians. We collected qualitative and quantitative data to 1) describe pig farms, 2) identify barriers to pig farming in Hong Kong and 3) describe the perceptions of the new service. Thematic analysis was conducted to identify barriers and attitudes.

**Results:**

Eight and nine out of 38 pig farmers agreed to participate in the qualitative and quantitative components, respectively. All farms were farrow-to-finish farms with a median of 2800 (range 950 to 7000) pigs per farm. Three themes were identified during the interview analysis and could be ranked based on their importance to the farmers: the regulatory environment (Theme 1), veterinary support structures (Theme 2), and the sustainability of the pig industry (Theme 3). Farmers expressed dissatisfaction with the regulation of the industry and veterinary services on offer within Hong Kong. However, farmers did note that the provision of a new pig health and production management service was as a positive development. The public perception of pig farming, market forces, and competition from mainland pig farmers have resulted in sustainability challenges for the industry.

**Conclusions:**

Farmers identified very specific local systems and challenges unique to pig farming in Hong Kong. The lack of veterinary support was one of these challenges and although a certain level of scepticism towards the new pig health and production service was expressed, farmers indicated their interest and listed areas where they would benefit from improved veterinary support. Prior experiences of veterinary services clouded farmers perceptions of the usefulness of a new service. To be successful in this environment, clear communication about the goals, role and limitations of the new on farm service is crucial, as is the alignment with the needs of farmers. Despite the small sample size, the qualitative methodology used allows us to assume that these themes give a general idea of what Hong Kong farmers’ concerns and attitudes are.

**Supplementary Information:**

The online version contains supplementary material available at 10.1186/s12917-023-03591-7.

## Background

Livestock veterinary services are important for endemic, trans-boundary and emerging disease prevention, treatment and management, food safety, animal welfare and improved production efficiency [1, 2]. Globally an increasing emphasis is being placed on prevention rather than the treatment of livestock diseases. Preventive measures include the promotion of husbandry practices with enhanced biosecurity and high animal welfare standards [2–5]. Disease prevention measures in pig production are more important than ever given e.g., the growing threat of African swine fever in Asia and globally [6–8]. The increased use of antimicrobials and the potential for the development of antimicrobial resistance in livestock production or the wider environment is another area of concern of veterinary and public health importance [9], and has also been associated with poor welfare and biosecurity practices [10–12]. Furthermore, pigs can be potential reservoirs of several zoonotic diseases that can compromise food safety and public health [13, 14]. It is therefore clear that a good animal health status and high welfare standards are indispensable for safeguarding food safety, improving farm productivity and ensuring sustainable farming. In Europe and North America, veterinarians are an integral part of the pig farming system, with a focus on the role of advising farmers on animal health and management, and welfare issues [15–18]. However, the use of veterinary expertise to maintain and improve health, productivity and welfare is not commonplace worldwide.

Hong Kong is an urbanised special administrative region within China, with a heavy reliance on the importation of food. Hong Kong does have a small commercial farming industry, producing pork, poultry, and aquaculture for the domestic market. In total, domestically produced pigs account for approximately 14% of all pigs slaughtered in Hong Kong, with the majority imported from China. In 2019, there were 40 active pig farms owned by 38 pig farmers, producing a total of approximately 100,000 pigs per year [19–21]. In Hong Kong, all owners of a domestic pig farm have to hold a valid licence issued by the government to produce pigs for slaughter. As part of the licence conditions, the authorities prescribe the maximum number of livestock that can be kept on the farm, as well as waste and biosecurity management measures for each farm. The licence has to be renewed every three years.

In Hong Kong, little is known about the pig farming system and unique challenges that the industry faces. Historically, veterinary involvement with the pork industry in Hong Kong has been limited to government veterinarians. This role included the control of imported pigs, veterinary pharmaceuticals and vaccines, the management of legislation, outbreak investigations, biosecurity advice, and diagnostic testing. The current study is part of a larger, government-funded project to enhance the economic and environmental sustainability of pig farming in Hong Kong (https://www.cityu.edu.hk/ohrp/ambulatory-veterinary-services/improving-pig-health-and-production-hong-kong-completed). This project was launched in 2018 and was led by City University of Hong Kong (City University). The aim of the project was to provide pig health and production management services for individual farms, tailored to the needs of Hong Kong’s pig farming industry, and provided by a veterinary team.

As the new pig health and production service was not a farmer-led initiative, it is of importance to identify how it would add value to farmers and what pig farmers would expect from such a service or what would be required to meet their needs. The uptake would depend on a variety of factors, including farmer demographics, problem awareness, openness to change, and sociocultural norms [18, 22–24]. Additionally, the ability or likelihood of farmers to engage with veterinarians to improve animal health, management, welfare and productivity will depend on their perceptions of the usefulness of such a service and the general issues faced by the farming community. Without accounting for farmers' perceptions of what could be offered, or their current broader constraints with regards to their business, interventions to improve pig health may be ineffective, lead to resentment or face outright rejection by farmers. A good relationship between veterinarians and farmers is an essential prerequisite for developing a meaningful understanding of a farming system, and to implement interventions that are more likely to be effective and sustainable [25–28]. Therefore, the aim of the current study was to identify barriers and attitudes of farmers associated with the provision of pig health and production management service in Hong Kong. This study was conducted alongside the implementation of the new service. The objectives were to 1) describe pig farms and farmers, 2) identify barriers to pig farming and 3) describe the perceptions of the new pig health and production management service. To address these objectives, we used both quantitative and qualitative approaches to collect data during face-to-face interviews with pig farmers.

## Results

The data collection for the research took place from 6 August 2019 to 28 September 2019. The in-depth interviews took between 45 to 90 min and were conducted in public venues like cafes.

### Participant characteristics

Nine pig farm owners participated in the study, thus the response rate was 24%. One of them refused to complete the questionnaire. All of the participants were male, with their demographic characteristics described in Table 1. The median length of farming experience farmers had was 22.5 (range 5 to 55) years. All participants were members of the Hong Kong Livestock Industry Association (HKLIA). Five participants had good or excellent English proficiency in reading and writing. Two participants could only read and write in Cantonese.

**Table 1:** Demographic characteristics of eight participants and their farms included in the study. The data were collected during an interview conducted between August and September 2019 with Hong Kong pig farmers

Variable	Category	Count (percentage)
Age (years)	21–30	1 (12.5)
	31–40	2 (25.0)
	41–50	1 (12.5)
	51–60	1 (12.5)
	> 60	3 (37.5)
Education	Primary	1 (12.5)
	Junior secondary school	2 (25.0)
	Senior secondary school	1 (12.5)
	College/ university	3 (37.5)
	Postgraduate degree	1 (12.5)
Owner of farmland	Farmer	4 (50.0)
	Government	1 (12.5)
	Landlord	1 (12.5)
	Government and landlord	2 (25.0)

The farms were farrow-to-finish operations, with a median of 2800 (range 950 to 7000) pigs per farm. Farmers reported sending a median of 348 (range 80 to 480) pigs to slaughter each month. Six (75%) of the farmers decided which pigs were to be slaughtered, one farmer relied on the decision of the pig trader and for one farmer it was a joint decision. All farmers reported that their last pig purchase were pigs sourced from Taiwan (Supplementary Table 1); previous pig purchases were sourced from Taiwan, China and the United States of America and the median number of purchased pigs was 58 (range 14 to 200). All farmers employed farmworkers from mainland China (median: 7.5 workers per farm; interquartile range: 3.5 to 9). Only three had also employees from Hong Kong.

### Thematic analysis

Three major themes were distilled from the transcripts of the participants. The themes described below are structured in a way that indicates the degree of importance the participants emphasized during the interview. This means that Theme 1 was considered the most important difficulty encountered, and Theme 3 was considered, relatively speaking, the least important one. Quotations from the raw data are used throughout the text to illustrate concepts within each category. To increase the clarity of the statements, some parts may be omitted or inserted by the authors. These omissions or insertions are indicated by square brackets.

#### Theme 1: Regulatory environment

Pig farmers identified infrastructure and licensing as constraints to the development of their business. When intending to make improvements to their farm infrastructure, pig farmers found it very time consuming having to seek approval from several government departments, each responsible for a different aspect (e.g., Lands Department, Environmental Protection Department, Fire Department, Transport Department, etc.). For example, one of the pig farmers said:

*“One of the biggest issues for this industry would be the land administration, since we are not allowed to make a lot of changes to the building structures of the pig farms. That’s why the pig farms in Hong Kong look just like chicken farms. Like those old days when farms were made of iron sheets with a piece of iron sheet on the top as the roof, and the pigs were raised underneath. Farmhouse. They call them agricultural structures. It can’t be larger than 1,000 sq. feet; the ceiling can’t be higher than 4.57 meters. Otherwise, you need to submit a plan for architecture. So, I consulted a lot of architects. […] Things just went complicated.”*

With respect to licensing, participants believe that the limited options associated with licence transfer are a constraint to the development of the industry. While the name of the licence holder can be changed, the location of the farm cannot be changed. One pig farm owner said the following:

*“Then other issues are caused by limited space here in Hong Kong and that is particularly notable in [… which is ] included in the government’s housing development plan […], which means that our current farm land will disappear. […] we have asked for alternative farm land, but the government showed little interest and offered no responses, in a typical bureaucratic manner.”*

Another issue mentioned by the informants related to the limited number of pigs allowed under each licence, and the restrictions imposed in relating to purchase of medication, especially antimicrobials, for their pigs.

#### Theme 2: Veterinary support structures

At the time of the interviews, pig health services were provided by both government and City University’s new service.

All interviewed pig farmers indicated that the existing veterinary services did not provide them with useful pig breeding and husbandry guidance which would allow the industry to improve productivity. One pig farmer elaborated:

*“If someone would show us [the farmers] how to improve our breeding and husbandry systems with [veterinary] support, then we [the pig industry] can increase farm productivity and performance. The problem is that in Hong Kong now there is no one that can teach [the farmers] how to do so.”*

In relation to engagement with pig farmers, it was mentioned that providers often were unable to deliver a timely service, veterinarians had limited understanding of the local industry, and did not seem to protect commercial secrets of individual pig farmers. In relation to a slow response by the pig health service, one pig farmer remembered an emergency. He said:

*“We [the farmers] rely on the pig health service provided by the government. Whenever we report sudden death of pigs or any other emergency, they always send vets. But it takes time. And, when we deliver dead pigs to the laboratory for diagnosis, it often takes one to two weeks for us to receive initial results. A report can even take three to six months!”*

Participants generally expressed appreciation in relation to City University’s service as they believed that it would contribute towards improving the professional standard of the industry. However, they pointed out that the new service was not very useful to them yet because the new veterinarians did not speak the local language and lacked the specific understanding of local pig production. For example, one pig farm owner said:

*“We have got to where we are today as farmers purely through learning from our own experiences, we have learnt by doing. We have reservations about using CityU’s [veterinary] services. It is difficult for us to put faith in textbook [theoretical] and overseas gained knowledge, as most veterinarians from CityU seem to offer this, […] rather than Hong Kong based experience. We are facing unique constraints within Hong Kong, with the shortage of land and space. [….] it is impossible to change our reservations on vets [from CityU], as they have limited experience with [local] pig farming […]. In comparison to their exposure to domestic pets, typically cats and dogs, which they usually handle on a daily basis; they have next to no contact or handling experiences with [local or mainland] pigs. We [pig farmers] are looking after thousands of pigs on daily basis. Well, it is not hard for you to understand our reservation.”*

Some pig farmers also mentioned the lack of electronic health and production recording systems on their farms. One pig farmer expressed its importance as follows:

*“Being able to gather and provide a pool of data and references to the industry is much more effective than the current seminars on offer.”*

Another pig farmer further elaborated with respect to the impact of not having electronic health and production recording systems, he explained:

*“Because no one is monitoring [the farmers’] progress on raising pigs in Hong Kong. For example, how many piglets each sow gives birth to, or like the exact number of pigs on the farm […]. No one has data like this. […] You don’t even have a set of data for the vet to do a proper diagnosis, then how would they know exactly what problem you are having? So, this is the case, let me tell you. The vet comes to your farm and he walks around. What he sees are the things that are happening on that very day.”*

#### Theme 3: Sustainability of pig industry

All pig farmers saw problems in actively sustaining the pig industry in Hong Kong. Specifically, these problems included a lack of understanding of the industry among the general public and local neighbours, labour shortages, a lack of research and development (R&D) support for long term development and strong competition from mainland pig farmers.

The first two problems were described as interrelated issues, with participants pointing to the lack of information about the industry, not to mention, the lack of educational courses about pig farming in Hong Kong society. Such a lack of understanding had created a negative sentiment of the industry among the general public as the conflicts between pig farmers and their residential neighbours were not uncommon. Farmers described situations where local people generally did not welcome the presence of a pig farm in their neighbourhood. This was mainly attributed to the bad odour and waste management associated with pig farming. Participants explained that since local Hongkongers did not know much and had already established a negative image about the industry, they were unwilling to join. Due to labour shortages on farms, workers needed to be hired from elsewhere, particularly from mainland China. But this resulted in concerns from participants that this would further tarnish their image with the public, as the public perception is that mainland workers take away work opportunities for local people.

For “lack of R&D support for long term development”, most informants mentioned their concerns about the lack of professional pig equipment companies to provide or develop hardware or technology for their industry in Hong Kong. For example, the participants mentioned the lack of i) computerized systems that could track pig’s physical conditions over time; ii) professional formula for pig’s food; and iii) specific hardware systems that supported local pig farming in managing ventilation and sewage. While these support tools are available elsewhere, it is hard for the Hong Kong farmers to find material and companies suitable for their needs locally. To import materials is expensive and laborious. For example, not all farmers would be able to speak languages other than Cantonese or Mandarin to order online.

Regarding the lack of computerized monitoring systems, one of the pig farmers mentioned or assumed that the problem was very common in Hong Kong. He said:

*“I believe there are very few of us in Hong Kong, maybe only 2 or 3 including me, actually have [pig’s] performance data. Because no one is monitoring their progress on raising pigs in Hong Kong. For example, how many piglets each mother pig gives birth to, or like the exact number of pigs on the farm is [ ]. No one has data like this. All these are basics for pig farmers overseas. They computerize their systems. For example, every time a pig gives birth, they will enter that data into the computer, then run data analysis on the farm after one year. By doing so, they know exactly how well their farms are doing, or how badly. In Hong Kong, I believe there is no one doing this [ ].”*

For the problem of strong competition from mainland pig farmers, participants noted mainland pig farmers have a much higher local market share in Hong Kong of between 90 and 95%. Mainland farmers also sell their pigs at lower prices, when compared to local farmers. Given this situation, local pig farmers have very limited negotiating power in relation to price. Facing critical external challenge within a small sector, Hong Kong pig farmers found it difficult to stay united. Participants expressed that there was little interest in collaboration with each other due to the competitive environment. One reason was that pig farmers did not have a shared vision for the future of the industry, as one participant elaborated:

*“There [is] great potential [ ] in pig farming in Hong Kong, but there are too many different voices and directions within the industry, too divided. As discussed earlier, every farmer takes [his] own approach, for example everyone uses different brands of vaccine with different procedures. There are also some who see [ ] no future for the industry and are unwilling to invest or progress.”*

## Discussion

Our study has been the first to explore the pig farming industry and to identify barriers to farming in Hong Kong. Early in the implementation of a new pig health and production management service, we sought to understand pig farmers’ perceptions of, and attitudes towards a veterinary service. The qualitative approach allowed for the development of a baseline understanding of pig farming in Hong Kong and to obtain insight into the issues faced by farmers and the utility of a veterinary service, the type of which was not previously available to farmers in Hong Kong. We identified three themes during the interviews with farmers, two industry-related and one specifically about veterinary services in Hong Kong. While the current situation was perceived negatively by farmers, the provision of a new pig health and production management service was seen as a positive future development. Despite the overwhelming difficulties articulated by the participants, it should be noted that strategies to address these challenges were also identified. Farmers were actively seeking to address challenges at the time of interview, as well as taking a future focused approach to enhance pig farming productivity and business viability.

Although the focus of the study was to identify barriers and attitudes of farmers associated with the provision of pig health and production management services, the main barrier that the farmers identified was in relation to the constraints imposed by government regulation for pig farming in Hong Kong. Farmers felt that improvement in on-farm infrastructure and pig husbandry practices was often hindered by outdated regulations, and they felt they had limited ability to influence review of these regulations. This finding highlights opportunities for the government and a veterinarian run service to partner with farmers to improve standards. By the government allowing the updating of farming infrastructure and capacity, the farmers will be able to improve animal health, welfare and productivity. But it will also be important for farmers to accept that the government implemented these regulations to serve the needs of the highly urbanised population of Hong Kong.

Within Theme 2, farmers commented that they felt that the existing private veterinary service providers in Hong Kong were mainly targeted at domestic pets, and therefore the training of veterinarians focused on this area, rather than on pig farming. Additionally, farmers felt reluctant to engage with the veterinary support services provided by the government, due to prior negative experiences. This perception by farmers of such service providers has been previously identified in other livestock industries and countries in response to feeling sceptical about advice provided [29] or actions taken during a crisis [30]. While in other countries, the relationship between on-farm veterinarians and farmers would be seen to bridge this gap [31], this is not the case in Hong Kong, where on-farm livestock veterinarians are not presently available. To be successful in this environment, clear communication about the goals, role and limitations of the new on-farm service is essential, and these should be aligned with the needs of farmers [32].

The sustainability of pig farming in Hong Kong was a barrier identified in Theme 3, where threats external and internal to the industry were identified. Participants identified elements linked to the three pillars of sustainability: economic, socio-cultural and environmental [33]. From a business perspective, local livestock producers must survive in a highly competitive economic climate where product safety, quality and price need to be kept in a very dynamic balance. Maintaining economic viability was identified as a challenge by farmers, as they could not produce pigs as cheaply as those coming from the mainland, nor had they found a mechanism to sell their product at premium prices. Within the socio-cultural pillar, most of Hong Kong’s citizens had a limited understanding of how their food is produced and showed little tolerance for sharing their environment with pig farms, in part due to being a highly urbanised society. Socio-cultural factors contributed to why there were very few local farmworkers. Farmers highlighted the environmental pillar as a barrier to sustainability within Theme 3 and Theme 1. Improving access to technology was one way farmers identified to improve the economic pillar through increased productivity and the environmental pillar through more efficient waste management. Improvements in productivity and efficiency are predicated on data collection, and information specific to a farm can help to drive change.

For a pig health service to be functional, there needs to be a balance between the needs of the farmer and what is possible to provide by a veterinarian run service. In Hong Kong, both farmers and veterinarians will need to take responsibility in building a sustainable, operational partnership. Based on the lack of unity within the industry, the farmer-side of the partnership may have to be developed on a case-by-case basis, rather than attempt to engage industry-wide. In practice, the development of a partnership may face difficulties as farmers expressed dissatisfaction with foreign-trained, non-local and non-native speaking veterinarians, who they perceived not to understand the challenges of farming in Hong Kong. Currently, there are no veterinary graduates trained in Hong Kong. Globally few veterinarians enter pig practice, limiting the supply of pig veterinary practitioners [34–36], and therefore even more in the local farming context.

Purposive sampling methods used as part of a qualitive methodology are an effective way to engage with communities in a research setting and provide an efficient and effective method to gain first insights into these communities [37]. The qualitative approach of semi-structured interviews enabled data collection to be driven by issues that were of importance to the farmers [38], further developing relationships through perception and recognition of the farmers’ perceived needs. The identification of themes and continuing purposive sampling until reaching information saturation are recognised methods to identify the perceptions and attitudes of participants [39–41]. The sample size was determined based on a saturation approach [42] and nine out of the 38 pig farmers in Hong Kong were interviewed. This approach impacted on the quantitative element of the study: the description of the demographics of pig farmers and the basic structure of their farms. As such, care should be taken when interpreting the quantitative data, as they may not be characteristic of all Hong Kong pig farmers. Farmers were predominantly male, covered various age groups (with a focus on people over 50 years of age), with differing education levels; demographics that largely represent local farmers. However, the median herd size of 2800 pigs (across all age categories) per farm is larger than the median herd size of all Hong Kong pig farms (of approximately 1300 pigs; personal communication). In this study, five herds were larger (data not shown). Such herds (and their farmers) might represent the trend to larger, more specialised herds. Additionally, we identified that only half of the farms belonged to the farmers. This can complicate decisions and investment in the future and could pose another challenge, both for farmers and for the new service.

In the current study, no analytical software was used to support the thematic analysis. While we have experience using qualitative analytical software [43, 44], there were several reasons why supporting the analysis using software was inappropriate here. Software is useful for analysing English-written or spoken texts, which are by nature systematically structured, and the meanings different actors (i.e., the author of the text) endow to the same keyword are relatively consistent and coherent. However, the application of software to the language of Chinese is a totally different case. In the current study the participants were speaking Cantonese (a language used mainly in southern China) of which many words uttered cannot be properly written in Chinese. Moreover, the participants were mainly older people with low educational level. The way the participants spoke Cantonese, and the use of words, were not the same as university students or formal situations. To check the consistency of interpretation and add robustness to the analysis, all of the interviews were transcribed into Cantonese (not Chinese) and English. This enabled exploration of plausible interpretations by the whole research team in the thematic analysis. It was clear through this iterative process that the sole reliance on the English transcription would have lost some of the richness within the information. This study highlights the importance of a pragmatic approach to qualitative data analysis.

As we did not speak to all 38 farmers, it is not known whether the opinions presented here are representative of all Hong Kong pig farmers. One of the limitations of the current study was farmer willingness to engage with researchers. To facilitate engagement, the researchers used a gatekeeper approach; the interview team was independent from both the new service and the government; and interviews were conducted in Cantonese, the native language of the interviewers and participants. Despite these measures, some pig farmers may have been less willing to participate. Within qualitative research, groups that are harder to reach can have different characteristics to study participants, with particular strategies emphasising engagement with these groups [45]. In the current study, it is hypothesised that these farmers would be more likely to show an unwillingness to change farming practices, while the farmers who are already engaging with the service, or participated in the current study, were those who were already looking or open to the idea of behavioural change. Future work with pig farmers in Hong Kong should actively seek to engage with this hard-to-reach group; however, success will be limited until issues raised by the participants regarding the utility of a pig health and production management service have been addressed. Nevertheless, based on the sentiments identified in the current study which was conducted at a time when the pig health and production service was newly established, pig farmers who are future focused, and those who have engaged with this study, but also some of the harder to reach farmers, are positive regarding the future value of a tailored, functional service for their pigs.

## Conclusions

This study explored pig farmers’ perceptions of, and attitudes towards the development of a pig health and production management service in Hong Kong, early in the implementation of a new service. It identified challenges for the development of pig production in Hong Kong. Farmers mentioned three main themes, ranked in order of importance: constraints imposed by the regulatory environment, unsatisfactory veterinary support structures, and sustainability challenges for the industry. The pig production system in Hong Kong is likely to differ markedly from other places in the world, because it is located in a relatively small geographical territory shared with a highly urbanised human population. The combined influence of public perception, market forces, government regulation and veterinary support have resulted in very specific local system characteristics. But it also needs to be recognised that the increasing global focus on One Health, food security, sustainability and animal welfare highlights the need for pig farmers to work more effectively with pig health and production management services to achieve the required production standards. To achieve that goal, it is of critical importance to provide a service that is tailored to the local pig production system and trusted by the pig farmers.

## Methods

### Sampling frame and study design

To address the objectives, the current study took a mixed methods approach to collect data during face-to-face interviews with pig farmers. The study design for the qualitative component was a case study [46, 47]. The study design for the quantitative component was a survey. The sampling frame consisted of all pig farms in Hong Kong, which must be registered with the Agricultural, Fisheries and Conservation Department (AFCD) to keep pigs for commercial purposes in 2019. The list contained 43 farms, with 38 farmers actively keeping pigs in 40 farms at the time of the survey. All farms were in the New Territories area of Hong Kong and supplied slaughter pigs for the domestic market. Most pig farmers in Hong Kong are members of the Hong Kong Livestock Industry Association (HKLIA), a group that organises regular meetings for farmer gathering, discussion and decision making.

### Sampling and data collection

The sampling strategy was purposive and relevant to the topic under investigation [38, 48]. This strategy enabled the selection of participants based on willingness to participate, and to canvas a range of opinions of importance to the farmers [38].

As this population had not been approached before, and there was no established relationship between researchers, veterinarians and farmers, the gatekeeper approach [49] was necessary to gain trust [50] and as a tool to support the sampling strategy. A gatekeeper was identified by researchers as a key member of the pig farmer section of the HKLIA. The gatekeeper was a trusted member of the group and was able to facilitate access to other pig farmers. Recruitment of participants through the gatekeeper occurred at a HKLIA meeting, with farmers followed up for interview after expressing interest in participating at the initial meeting. Farmers with established relationships with City University were approached directly.

In the current study, sampling was considered to be complete based on the notion of saturation [42] and the comprehensiveness off the information collated rather than sample size [51]. This was an iterative process, based on grounded theory and theoretical sampling [42], and evaluated the responses of each participant following interviews based on identified themes or categories. The number of participants interviewed was determined based on the consistency of answers. The sampling process was terminated when interviews did not generate any further information within each theme or category, e.g., a participant did not add additional knowledge to what had already been gathered from previous interviewees in relation to the identified themes. It was anticipated that there would be different types of farmers that could be recruited in the study; those already engaging -, those with an interest in engaging-, and those that are unlikely to engage with external veterinary services. Attempts were made to recruit participants from all three types of farmers. At the outset, it was noted that the size of the pig farming industry in Hong Kong was small. Despite this, the number of participants was expected to be small in order to support the depth of inquiry that was necessary and fundamental to qualitative research [38, 52, 53].

Ethics approval was given by the Human Subjects Ethics Sub-Committee of the City University of Hong Kong. Participants were informed about the goals and the methods including guaranteed anonymity before the interviews.

### Questionnaire design

The survey consisted of a two-page questionnaire and a semi-structured interview.

The questionnaire covered demographic and farm-level information. It was pilot tested with one farmer. The interview guide for the semi-structured interview consisted of four parts: Part 1: General Difficulties; Part 2: The New Service; Part 3: Business Process and Structure and Part 4: Future and Sustainability, with the questions for each section summarised in Supplementary Table 2. The interview guide was also pilot tested with one farmer and, after slight modifications, used throughout the following interviews to ensure consistent interviews.

All interviews were conducted by three trained interviewers who were independent from the government and independent from the new service, although they were employed by City University of Hong Kong. All interviews were conducted in Cantonese. The interviews were recorded with the participants’ agreement. To maintain the confidentiality of the participants, pseudonyms (Informant #1 to #10) were used when analysing the data.

### Data analysis

Descriptive characteristics of the participants collected in the questionnaire were summarised using number and percentage for categorical variables and median and range for continuous variables [54].

Thematic analysis was conducted on the audio files solicited from interviewing the nine participants [55]. The audio files were transcribed first in Cantonese (the language used during the interviews) and then translated into English by a professional translation service. The accuracy of both versions was checked carefully to make sure that the audio record and the two written documents were highly congruent. Specifically, all transcripts were reviewed by a bilingual research team member to ensure the accuracy of note-to-typed word translation. All interviews were reviewed, coded and categorised based on similarities between ideas and themes [39–41]. Initially, two researchers examined the data in Cantonese, with continual reanalysis throughout the analysis cycle. Major themes were distilled based on themes that were articulated prominently and repeatedly in the participants’ accounts. Secondarily, the English transcripts were examined to identify the themes and any divergence in meaning between the original Cantonese and English translation. Themes were then re-examined in English, with discrepancies in interpretation discussed and resolved by the research team. As such, the analysis focused on describing the sentiments of the participants, rather than quantifying the frequency of occurrence, although the importance of the themes to the participants has been noted. All the data presented in the results section reflect the observations, insights and opinions expressed by participants.

## Supplementary Information

**Additional file 1: Supplementary Table 1.** Additional categorical and continuous demographic characteristics of eight participants and their farms included in the study. The data were collected during an interview conducted between August and September 2019 with Hong Kong pig farmers.

**Additional file 2: Supplementary Table 2.** Topics and questions included in semi-structured interviews conducted with Hong Kong pig farmers to identify the barriers in pig industry and expectations from a pig health and production management service in autumn 2019.

## Data Availability

The corresponding author (SR) can be contacted for information regarding the dataset. However, the datasets generated and/or analysed during the current study will not be made publicly available to maintain anonymity of our participants, and to uphold conditions relating to informed consent.

## Abbreviations

AFCD: Agricultural, Fisheries and Conservation Department
City University: City University of Hong Kong
HKLIA: Livestock Industry Association
R&D: Research and Development
