# Supplementary material for: The challenges of pig farming in Hong Kong: a study of farmers’ perceptions and attitudes towards a pig health and production management service
Source: BMC Vet Res. 2023 Feb 1;19:30. doi: 10.1186/s12917-023-03591-7 (PMC9890852; doi:10.1186/s12917-023-03591-7)
Supplement: Supplementary file 1 — Additional file 1: Supplementary Table 1. Additional categorical and continuous demographic characteristics of eight participants and their farms included in the study. The data were collected during an interview conducted between August and September 2019 with Hong Kong pig farmers. [file 12917_2023_3591_MOESM1_ESM.docx]

**Supplementary Table 1**: Additional categorical and continuous demographic characteristics of eight participants and their farms included in the study. The data were collected during an interview conducted between August and September 2019 with Hong Kong pig farmers.

| Variable | Category | Median (range); number of answers |
| --- | --- | --- |
| Breed of pigs  (% of the total herd) | Large white  Duroc  Landrace  Landrace x large white  Landrace x large white x duroc  Black pig | 1 (0-40)  0 (0-5)  3 (0-40)  50 (0-97)  55 (22-88.5) (n=7)  15 (0-75) pig (n=1) |
| Size of three age categories (%) (n=7) |  | 5 (0-39) Gilts  77 (10-94) Sows  3 (0.3-10) Boars |
| Last purchase of pigs   - Number - % male/ female - Source of pigs |  | 58 (14-200)  2 (0-33)/ 98 (67-100)  100% Taiwan |
| Second last purchase of pigs   - Number (n=6) - % male/ female (n=5) - Source of pigs |  | 33.5 (20-60)  0 (0-1)/ 100 (99-100)  Taiwan (n=4), China (n=1), USA (n=1) |
| Number of slaughtering occassions last month (n=7) | | 25 (4-30) |
| Number of slaughter pigs last month | | 300 (70-800) |
| Factors for decision to send pigs to slaughter  Priority 1  Priority 2  Priority 3  Priority 4 | | Body weight (n=6) >> market demand (n=1) or market price (n=1)  Market price (n=4) > body weight (n=2) or marked demand (n=2)  Marked demand (n=5) >> health of the pigs (n=2)  Health of the pig (n=4) >> slaughter price (n=1) |
| Acquisition of pig farm (n=7) | | Inherited (n=4)  Newly established (n=2)  Partnership (n=1) |
| Number of generations (inherited farm) | | Third (n=2)  Second (n=2) |
