# Supplementary material for: The challenges of pig farming in Hong Kong: a study of farmers’ perceptions and attitudes towards a pig health and production management service
Source: BMC Vet Res. 2023 Feb 1;19:30. doi: 10.1186/s12917-023-03591-7 (PMC9890852; doi:10.1186/s12917-023-03591-7)
Supplement: Supplementary file 2 — Additional file 2: Supplementary Table 2. Topics and questions included in semi-structured interviews conducted with Hong Kong pig farmers to identify the barriers in pig industry and expectations from a pig health and production management service in autumn 2019. [file 12917_2023_3591_MOESM2_ESM.docx]

**Supplementary Table 2**: Topics and questions included in semi-structured interviews conducted with Hong Kong pig farmers to identify the barriers in pig industry and expectations from a pig health and production management service in autumn 2019.

| Topic | Questions |
| --- | --- |
| Part 1: General difficulties | 1. For the past few years, what are your difficulties in managing your pig farm? It can be the most troublesome, most challenged, most difficult from your experience. Please tell us your stories. 2. Which is the most difficult situation that you have encountered in your career? |
| Part 2: Pig health and production management service provided by veterinarians | 1. Where do you get information on pig health, such as diseases, treatment, vaccinations, etc.? 2. Have you had any experience(s) with any veterinary service with your pigs before? 3. Can you tell me some of the most memorable stories? |
| Part 3: Business Processes and Structure | 1. Can you describe the pig production cycle in your farm? 2. What are your difficulties in managing the pig production cycles? 3. Are there any competitors? 4. Are the government policies helping out the industry? Why or why not? |
| Part 4: Future and sustainability | 1. How did you become a pig farmer? 2. Why did you decide to be a pig farmer? 3. How did you get your knowledge and skills on pig farming? 4. Where do you see yourself as a pig farmer in ten years? 5. What is your opinion on the future of the local pig production? 6. What do you think Hong Kong pig farming will be like in the future? |
